# Transcranial Magnetic Stimulation in Psychiatry: Is There a Need for Electric Field Standardization?

**DOI:** 10.3389/fnhum.2021.639640

**Published:** 2021-03-09

**Authors:** Zsolt Turi, Claus Normann, Katharina Domschke, Andreas Vlachos

**Affiliations:** ^1^Department of Neuroanatomy, Institute of Anatomy and Cell Biology, Faculty of Medicine, University of Freiburg, Freiburg, Germany; ^2^Department of Psychiatry and Psychotherapy, Medical Center—Faculty of Medicine, University of Freiburg, Freiburg, Germany; ^3^Center for Basics in NeuroModulation (NeuroModulBasics), Faculty of Medicine, University of Freiburg, Freiburg, Germany; ^4^Center Brain Links Brain Tools, University of Freiburg, Freiburg, Germany

**Keywords:** non-invasive brain stimulation, repetitive transcranial magnetic stimulation, motor threshold, electric field modeling, depression

## Abstract

Single-pulse and repetitive transcranial magnetic stimulation (rTMS) are used in clinical practice for diagnostic and therapeutic purposes. However, rTMS-based therapies that lead to a significant and sustained reduction in neuropsychiatric symptoms remain scarce. While it is generally accepted that the stimulation frequency plays a crucial role in producing the therapeutic effects of rTMS, less attention has been dedicated to determining the role of the electric field strength. Conventional threshold-based intensity selection approaches, such as the resting motor threshold, produce variable stimulation intensities and electric fields across participants and cortical regions. Insufficient standardization of electric field strength may contribute to the variability of rTMS effects and thus therapeutic success. Computational approaches that can prospectively optimize the electric field and standardize it across patients and cortical targets may overcome some of these limitations. Here, we discuss these approaches and propose that electric field standardization will be instrumental for translational science frameworks (e.g., multiscale modeling and basic science approaches) aimed at deciphering the subcellular, cellular, and network mechanisms of rTMS. Advances in understanding these mechanisms will be important for optimizing rTMS-based therapies in psychiatry.

## Introduction

Transcranial magnetic stimulation (TMS) is a non-invasive brain stimulation (NIBS) method widely used in neuroscience research and clinical practice (Huang et al., 2017; Bergmann and Hartwigsen, 2020). Based on the physical principle of electromagnetic induction, TMS produces short (~200–500 μs) but strong (>1.5 Tesla) magnetic fields that penetrate the intact skin and skull of patients. Hence, TMS produces its major effects by inducing peak absolute electric fields (~100 mV/mm) in cortical brain regions (Paulus et al., 2013).

When applied repeatedly (i.e., ≧1 Hz), repetitive TMS (rTMS) induces lasting changes in cortical excitability and plasticity, making rTMS a suitable tool for modulating complex brain function in health and disease (Lefaucheur et al., 2014; Huang et al., 2017). Recent research has demonstrated that rTMS is capable of inducing long-lasting plasticity of excitatory and inhibitory neurotransmission in animal models (Gersner et al., 2011; Ma et al., 2013; Lenz et al., 2016, 2020; Tang et al., 2017).

TMS was first introduced in 1985 (Barker et al., 1985), and the first rTMS study was performed in 1991 in epileptic patients (Pascual-Leone et al., 1991). Four years later, the first rTMS study in psychiatric patients suffering from depression was published (George et al., 1995). Since then, rTMS has been used to treat various neuropsychiatric conditions associated with alterations in cortical excitability, including movement disorders, Alzheimer’s disease, depression, anxiety disorders, obsessive-compulsive disorders, and schizophrenia (Lefaucheur et al., 2014).

**Figure 1:**
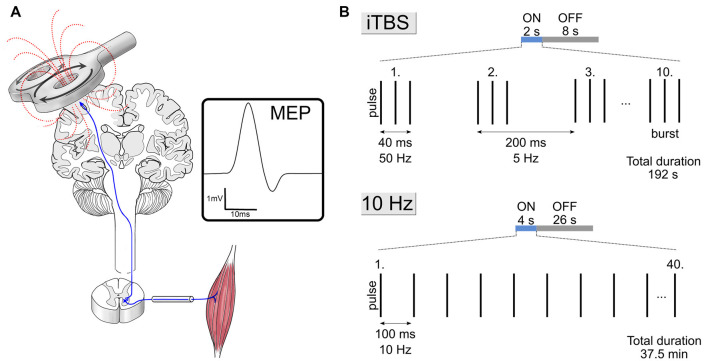
Single-pulse transcranial magnetic stimulation (TMS) and clinically approved stimulation protocols. **(A)** Visualization of TMS of the motor cortex while recording motor evoked potentials (MEP) of the target muscle. The intensity of TMS is adjusted by recording the amplitude of MEPs elicited from the target muscle while stimulating its motor cortical representation (see Vlachos et al., 2017). **(B)** Schematic illustration of stimulation parameters of the 10 Hz and intermittent theta-burst stimulation (iTBS) repetitive TMS (rTMS) protocols. Both protocols use stimulation intensities at 120% of the motor threshold.

Two rTMS protocols received approval from the U.S. Food and Drug Administration (FDA) for the treatment of pharmacoresistant depression; the 10 Hz rTMS protocol in 2008 and the intermittent theta-burst stimulation (iTBS) protocol in 2018 (Figure 1). Indeed, a previous meta-analysis including 81 studies (*n* = 4,233) revealed significant short-term antidepressant effects of rTMS (Brunoni et al., 2017). The most recent meta-analysis including ten studies (six randomized controlled trials, *n* = 294; four uncontrolled clinical trials, *n* = 297) on iTBS to the dorsolateral prefrontal cortex (DLPFC) vs. standard rTMS reported overall effect sizes for response and remission rates of 0.38 and 0.20 in depressed patients, respectively (Chu et al., 2020). Finally, a meta-analysis of eighteen studies on the sustainability of rTMS effects in depression showed sustained response rates of about 50% 3, 6, and 12 months after initial treatment (Senova et al., 2019). In sum, these meta-analyses point to a significant efficacy and durability of rTMS in the treatment of depression.

Still, the clinical relevance of rTMS for depression is debated. Whereas short-term effects in treatment-resistant depression have been repeatedly demonstrated, sustained efficacy regarding clinically relevant outcomes has been questioned (Lepping et al., 2014; Kedzior et al., 2015; Ontario, 2016; Papadimitropoulou et al., 2017). Moreover, a high placebo response, technical difficulties in obtaining a double-blinded sham response, and rather small sample sizes compared to large psychopharmacological trials seem to hamper the validity of several rTMS clinical trials (Razza et al., 2018). Apparently, a more reliable and sustained clinical efficacy of rTMS—based on reproducible neurophysiological effects—is urgently needed.

While it is generally accepted that the stimulation frequency plays the dominant role in producing the therapeutic effects of rTMS, less attention has been dedicated to the role of electric field strength. In this perspective article, we discuss the use of computational modeling to standardize the stimulus intensities by closely matching electric field characteristics in a given cortical target across the study sample. Such an approach could be instrumental for developing suitable translational research frameworks for obtaining insights into the biological and therapeutic mechanisms of rTMS effects in the treatment of neuropsychiatric disorders.

## Motor Threshold-Based Intensity Selection for rTMS

In human rTMS-studies, the vast majority of studies use the motor threshold (MT) approach for intensity selection (Turi et al., 2020). This approach adjusts the intensity of TMS by recording the amplitude of motor evoked potentials (MEPs) elicited from the target muscle while stimulating its motor cortical representation (Figure 1A). In research and clinical settings, typical intensities range between 80–120% of the MT for the stimulation of brain regions other than the motor cortex (e.g., the prefrontal cortex; Turi et al., 2020). For example, the FDA-approved 10 Hz and iTBS protocols (Figure 1B) both use 120% of the resting MT intensity for the stimulation of the prefrontal cortex (Blumberger et al., 2018).

One limitation of the MT approach is that the properties of the electric fields, both in the motor cortex and therapeutically targeted brain regions, remain undefined. Moreover, the physiological mechanisms of inducing MEPs by TMS are not completely understood in humans. For example, a crucial question pertains to the mechanisms of TMS activation of layer V pyramidal neurons (Di Lazzaro and Ziemann, 2013). These neurons form the corticospinal tracts, driving the activation of motor neurons in the spinal cord and subsequent muscle activation. It is currently unclear whether layer V pyramidal neurons are depolarized directly by TMS or indirectly *via* the stimulation of axons terminating onto layer V pyramidal neurons (Di Lazzaro and Ziemann, 2013). Likewise, the role of (in)direct activation of interneurons during TMS remains unclear.

MT intensities can vary substantially across participants, and studies frequently fail to report the stimulation intensity translated into physical parameters, such as percent of the maximum device output (Turi et al., 2020). In this context, it is important to note that the amplitude of MEPs is susceptible to attentional and voluntary mechanisms (Bell et al., 2018; Ruddy et al., 2018). Participants can be trained to significantly decrease or increase the amplitude of their MEPs voluntarily (Ruddy et al., 2018). Thus, intensities corresponding to a given MT are expected to yield distinct electric field strengths in different participants or even in the same participants under certain conditions.

Finally, the stimulation intensities estimated with the MT approach may vary substantially between studies depending on the type of the threshold (e.g., active or resting) and the exact procedure used for detecting the MT (e.g., visual vs. electrophysiological MEP detection). Hence, this conventional intensity selection approach cannot adequately standardize the electric field properties across participants and cortical regions. Thus, a remaining open question is whether the MT-based intensity selection approach can explain, at least in part, the considerable inter- and intra-individual variability of rTMS-induced aftereffects.

## Standardization of Stimulus Parameters in Basic Science Experiments

From a translational point of view, the stimulation intensity expressed as a given percentage of the MT (e.g., 120% resting MT), is not informative for basic science experiments aimed at deciphering the mechanisms of rTMS-based therapies. A major advantage of carrying out rTMS experiments in suitable animal models both *in vivo* and *in vitro* is the ability to readily standardize electric fields (and other stimulation parameters) across experiments. For example, in our *in vitro* experimental procedures, all stimulation parameters are kept constant, and the same target volume is stimulated in every experiment, i.e., brain tissue cultures in a standard 35 mm Petri dish filled with artificial cerebrospinal fluid (see Müller-Dahlhaus and Vlachos, 2013). Due to the standardized Petri dish volume, coil-to-tissue culture distance, and tissue culture size, this approach yields closely-matched electric fields in our laboratory.

Indeed, the effects of 10 Hz repetitive magnetic stimulation on synaptic plasticity *in vitro* are robust and highly reproducible (Vlachos et al., 2012; Lenz et al., 2015, 2016, 2020). It is important to emphasize, however, that *in vitro* preparations are not comparable to the complex *in vivo* situation and do not allow for a straightforward translation to treating the diseased human brain. Nevertheless, careful standardization of stimulus parameters—specifically, electric field strength and direction—seems mandatory for a systematic assessment of factors that may affect the outcome of a given (standardized) therapeutic rTMS protocol in suitable animal models (De Risio et al., 2020) as well as human rTMS studies.

## Importance of Electric Field Strength Standardization in Clinical Settings

The relevance of careful standardization of electric fields is also supported by studies using transcranial electric stimulation (tES; Antal et al., 2017); another clinically employed NIBS method. The two most frequent tES approaches use either direct (i.e., constant) or alternating (i.e., oscillating) currents for brain stimulation between two or more electrodes that are attached to the skin of the skull.

Studies using tES congruently suggest that the stimulation intensity can have a significant impact on the physiological aftereffects of the intervention. For example, Batsikadze et al. (2013) have shown distinct aftereffects of transcranial direct current stimulation (tDCS) applied at 1 and 2 mA intensities. The corticospinal excitability in humans was decreased by 1 mA cathodal tDCS, whereas 2 mA increased excitability (Batsikadze et al., 2013).

Similarly, Moliadze et al. (2012) demonstrated opposing effects of 140 Hz transcranial alternating current stimulation (tACS) at different intensities. While 0.6 mA decreased the level of corticospinal excitability, 1 mA increased it (corresponding to less than 0.2 mV/mm change in the absolute electric field in the motor cortex). These studies suggest that small changes in the electric field strength play a crucial role in inducing the physiological aftereffects of tES.

The electric fields induced by TMS in the human cortex are several-fold stronger than the electric fields achieved with tES or magnetic stimulation of the rodent brain *in vivo* and *in vitro*. Therefore, a systematic analysis of the dose-response effects—the role of the electric field strength in rTMS-induced (therapeutic) aftereffects—seems urgently needed. In this context, computational modeling has the potential to provide a translational framework for the physical input parameters of TMS, such as electric field properties, and neural responses in the human cortex and in suitable animal models.

## Computational Modeling for Estimating Electric Fields and Prospective Electric Field Optimization

**Figure 2:**
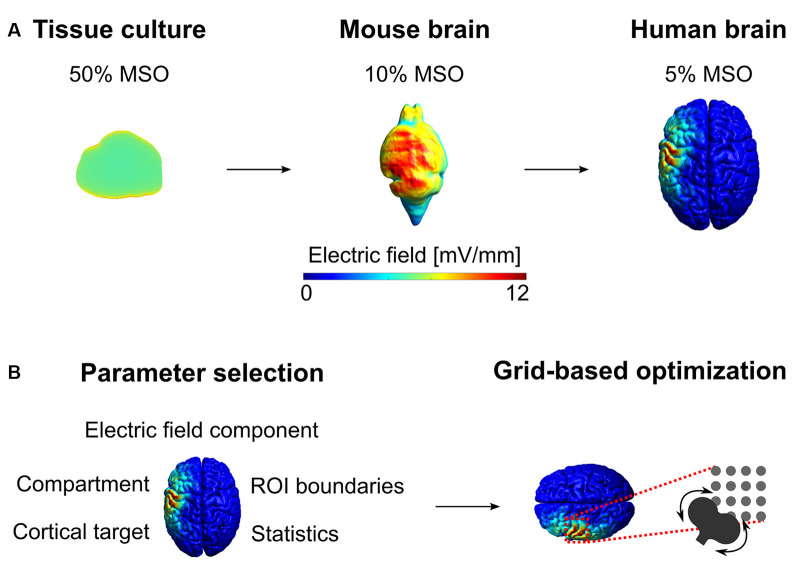
Electric field modeling of *in vitro* tissue culture and in the mouse and human brain. **(A)** First, we estimated the electric field in the tissue culture produced by the figure-of-eight TMS coil at 50% of the maximum stimulator output (MSO). Then, we calculated the robust maximum (i.e., 99.9th percentile) of the electric field distribution in the tissue culture and optimized the stimulation intensities to reproduce the same robust maximum in the gray matter volume compartment. Due to the large difference between the brain volumes, the stimulator intensity (expressed in MSO%) is substantially weaker for the mouse and human brain. The spatial distribution of the electric field intensities is more homogenous in the tissue culture with simplified geometry compared to the mouse and human brain. The mesh file for the mouse brain was obtained from Alekseichuk et al. (2019). **(B)** The choice of simulation parameters influences the characteristics of the electric field and the outcome of the optimization.

In recent years, sophisticated computer models have been developed to numerically calculate the induced electric fields in whole-brain volume conductor models (Figure 2). There are several, free toolboxes available for NIBS (Thielscher et al., 2015; Huang et al., 2019). These toolboxes can generate anatomically realistic, multi-compartment head models derived from structural magnetic resonance imaging data of the participants. The three-dimensional head models are generated by finite element or boundary element methods (Saturnino et al., 2019).

Electric field modeling seems to be particularly relevant when determining the stimulation intensity in cortical brain regions, such as DLPFC, that do not produce readily detectable responses, unlike the motor (e.g., MEPs) or visual (e.g., phosphenes) systems. In these cases, computational modeling can be used to adapt and standardize the stimulation intensities based on a prospective electric field optimization approach (Balderston et al., 2020; Beynel et al., 2020; Zmeykina et al., 2020).

After the preparation of the individual head model, several crucial simulation parameters must be considered for prospective electric field optimization (Figure 2B). To begin with, one needs to define the cortical target of the stimulation using one of the several approaches. These include; to define locations relative to the motor cortical representation of a given hand muscle (e.g., the 5 cm rule; Fitzgerald et al., 2009) or a probabilistic map of structural MRI (e.g., Montreal neuroimaging coordinates transformed into subject space; Blumberger et al., 2018). Other methods may use the location where phosphenes can be induced (i.e., occipital lobe; Brückner and Kammer, 2016), functional lesions (e.g., speech arrest; Pascual-Leone et al., 1991), or locations corresponding to electroencephalogram (EEG) electrodes (Zmeykina et al., 2020). Also, locations may be determined by magnetoencephalogram/EEG source analysis or fMRI functional localization at the single-subject or group level (Beynel et al., 2020; Zhang et al., 2020).

The electric field optimization may focus on the cortical surface or the volume compartment (Alekseichuk et al., 2019). Similarly, one may calculate the electric field properties at the entire compartment (e.g., gray matter volume) or on its subset, also called the region of interest (ROI). For ROI-based electric field analysis, one needs to specify its boundaries (e.g., it is surface and volume coordinates) and its shape (e.g., spherical, following the cortical folding pattern, et cetera). The choice of the ROI size will affect certain electric field properties. For example, around the same cortical target, the mean or median electric field strength will be weaker in larger ROIs (e.g., spherical ROI with 20 mm diameter) compared to a smaller ROI that elements are closer to the coil (e.g., 5 mm diameter spherical ROI).

Because the electric field is a complex, three-dimensional vector field characterized by its amplitude and direction, the characteristics of the electric field that will be considered in the electric field optimization process need to be determined. The electric field can be optimized for the absolute electric field strength or its normal (radial) or tangential spatial component for the cortical surface (Alekseichuk et al., 2019). Moreover, there are several ways to characterize the distribution of the electric field strength values in the ROIs, including calculating the robust maximum (usually between the 98th and 99.9th percentiles), mean, median, etc. values (Alekseichuk et al., 2019; Zmeykina et al., 2020).

The resulting electric field characteristics strongly depend on the coil center and its exact orientation. Therefore, a common procedure is to perform grid simulations on a series of predetermined coil locations while systematically manipulating the coil’s rotation angle around the coil’s center axis at each grid location. The outcome of this process is the most optimal coil location, orientation, and device intensity that produces the prospectively determined electric field characteristics in the ROI.

## Conclusion and Outlook

Despite careful standardization of the electric field strength, it is conceivable to assume that there is no single, universally effective electric field strength value. Instead, different electric field strengths likely induce different neural effects, perhaps even concurrently in a given cortical target (Liu et al., 2018). For example, weaker electric fields may only induce immediate effects, such as temporal shifts in neural spike timing, without inducing long-lasting aftereffects (Zmeykina et al., 2020). Protocols with stronger electric fields may reach the threshold for the induction of plasticity of excitatory or inhibitory neurotransmission (see Lenz and Vlachos, 2016).

Reverse translational approaches combining basic science methods with computational modeling can facilitate the identification of effective electric field strengths for different neuronal mechanisms. For example, one interesting approach may use standardized electric field values in a human neocortical target that has been demonstrated to exert specific aftereffects in an animal model. However, we have to concede that the link between the rTMS-induced cellular aftereffects and their therapeutic potentials in psychiatry remains unclear.

It is important to also emphasize that electric field estimation can provide only approximate values of the *de facto* electric field produced. Tissue segmentation inaccuracies, especially between the skull and cerebrospinal fluid can have a substantial effect on the estimated values. Computational modeling toolboxes require additional validation of the induced electric field for TMS. Therefore, one should interpret the exact electric field values with caution.

In summary, we propose that a more careful standardization of electric field strength in rTMS is instrumental for the optimization of current rTMS-based therapies in neuropsychiatric phenotypes. Prospective electric field simulations have the potential to provide a translational framework across distinct scales and experimental settings. Multiscale neuronal modeling of realistic rodent and human neurons provides a promising tool in rapidly screening distinct stimulus intensities, orientations, frequencies, and pulse numbers that can be validated in a translational approach for the optimization of rTMS-based therapies in psychiatry (Shirinpour et al., 2020).

## Data Availability Statement

The original contributions presented in the study are included in the article, further inquiries can be directed to the corresponding author.

## Author Contributions

ZT, CN, KD, and AV wrote the manuscript. ZT and AV prepared figures. All authors contributed to the article and approved the submitted version.

## Conflict of Interest

The authors declare that the research was conducted in the absence of any commercial or financial relationships that could be construed as a potential conflict of interest.
